# The challenges of hepatic epithelioid hemangioendothelioma: the diagnosis and current treatments of a problematic tumor

**DOI:** 10.1186/s13023-024-03354-z

**Published:** 2024-11-30

**Authors:** Manar Mikhail Atyah, Yongliang Sun, Zhiying Yang

**Affiliations:** Department of Hepatobiliary and Pancreatic Surgery, China-Japan Friendship Hospital, Beijing, China

**Keywords:** Hepatic epithelioid hemangioendothelioma, WWTR1–CAMTA1 fusion, Surgery, Anti-angiogenesis, Interferon, Sirolimus

## Abstract

**Background:**

Hepatic epithelioid hemangioendothelioma (HEHE) is a malignant vascular tumor known for its rarity. The different types of this hepatic tumor (single, multiple-nodular or diffused) indicate different prognosis and treatment plans. However, the heterogenic clinical manifestation creates a dilemma and a wide range of challenges when attending to HEHE patients. This review addresses the unique profile and clinical challenges that complicate the diagnosis and treatment of HEHE while focusing on current therapeutic strategies and their limitations.

**Main text:**

The unclear etiology is a challenging feature of HEHE. The exact involvement of potential risk factors and mechanism of development are still undefined. Relevant genetic alterations like WWTR1–CAMTA1 fusion have been investigated; however, they are only applicable as diagnostic markers and their influence on therapeutic efficacy is largely unknown. Other characteristics include asymptomatic manifestation, lack of unique hepatic functional alterations, high rates of misdiagnosis and late-stage identification when metastases already exist. Currently, tissue biopsy is the main tool to establish a definite diagnosis but is challenged with the limited awareness to suspect HEHE at early stages and the lack of relevant guidelines due to the rarity and the insufficiency of relevant research. The absence of treatment guidelines is the greatest challenge of HEHE. Generally, Surgical approaches are recommended due to the benefits of prolonged survival and enhanced prognosis. Nonetheless, only a minority of patients are eligible for resections while liver transplants are faced with severe insufficiency of donor organs and long wait-lists. On the other hand, a variety of non-surgical treatments (like anti-angiogenic agents, interferon alpha-2B and sirolimus) are presented with a promising potential. However, relevant studies are challenged with limited sample-sizes and lack of prospective designs.

**Conclusion:**

Regardless to decades passing since its discovery, HEHE still creates a dilemma due to its challenging clinical profile and lack of treatment guidelines. Raising awareness of HEHE in clinical practices improves the ability to diagnose this rare tumor at early stages and develop stronger research strategies and treatment guidelines to regulate the medical care provided to HEHE patients.

**Supplementary Information:**

The online version contains supplementary material available at 10.1186/s13023-024-03354-z.

## Background

Epithelioid hemangioendothelioma (EHE) is a rare malignant vascular tumor with an incidence of only 1/1000,000 [1]. Originating from pre-endothelial and endothelial cells of blood vessels [1], EHE has an epithelioid morphology with dendritic and endothelial cellular components [2]. Cytologically, EHE biopsy commonly includes a mixture of dispersed single cells with occasional presence of cellular aggregates, dense stromal and tissue fragments, epithelioid cells rich with dense cytoplasm, mild nuclear pleomorphism, nuclear grooves and occasional presence of intranuclear pseudo-inclusions and intracytoplasmic lumina [3]. Clinically, EHE incidence is influenced by gender and age with a predominance of mid-age (30–40) female patients [1]. Additionally, a striking heterogenicity is observed in the clinical manifestation of EHE including characteristics and site of tumor [4]. Although EHEs of the lungs and liver are common, cases of other sites (bones and soft tissues) have been reported. Thus, EHE can develop in different organs [5]. Such heterogenicity directly influences the prognosis and mortality rates which are higher for organs like the lungs and lower for EHEs in soft tissues [3]. 

As mentioned, the liver is a common site of EHE. Hepatic EHE (HEHE) is very rare [6] and shares EHE typical characteristics such as age and gender prevalence [2, 7, 8]. Clinically, HEHE includes single, multiple, and diffuse types with the majority of cases being multifocal [9]. As for malignancy, HEHE cases vary between low and medium grades [9] with a relatively better prognosis than other hepatic cancers. Generally, the 5-year survival rate of HEHE is 50% and the influence of metastasis on survival and treatment options is relatively limited when compared to other malignancies [10]. Nonetheless, HEHE creates a dilemma with its heterogenicity and wide range of challenges.

This review aims to address the unique profile of HEHE, discussing its presentation, unique diagnosis, treatment strategies and relevant limitations while shedding the light on the challenges limiting the quality and efficacy of clinical care provided to patients.

### The clinical presentation and diagnosis of HEHE

HEHE is known for its unspecific clinical manifestation and lack of early accurate diagnosis. Since single nodular HEHE is limited to early stages, the majority of cases are multi-nodular with a double-lobe involvement [7]. Studies reported that multi-nodular HEHE makes up to 66.5% of all cases with 82.2% involving both hepatic lobes [11]. As for the diffuse type, it is usually associated with late stages and accompanied with extrahepatic metastasis [9]. Regardless to the relatively low malignancy [9], metastasis is commonly encountered in HEHE. Earlier investigation by Läuffer et al. reported an over-all metastasis rate of 45.1% [12] while Mehrabi et al. observed a 36.6% extrahepatic metastasis rate involving the lungs, lymph nodes, peritoneum, bone, spleen and diaphragm [7]. For some cases, metastasis to several extrahepatic sites can be observed in a single patient. Duletić-Nacinović et al. [13] reported HEHE in a mid-age female patient with metastases to lungs, spleen, vertebrae, iliac bones and central nerve system. Other less-common sites such as myocardium [14], superior vena cava [15], omentum and mesentery [16] have also been reported.

In addition to the metastatic tendency, HEHE is also known for its asymptomatic clinical presence. Approximately, more than 25% of cases are asymptomatic [7] and 40% diagnosed incidentally [17]. Furthermore, symptomatic cases tend to be nonspecific and easy to be overlooked such as abdominal discomfort, nausea, weight loss, fatigue, or loss of appetite [9]. In addition, HEHE lacks any unique changes in liver function markers usually observed in other hepatic cancers. Such features led to a high rate of misdiagnosis (in up to 80% of cases) [7]. Thus, it is essential to clearly identify HEHE and differentiate this tumor from other malignancies like hepatocellular carcinoma (HCC), metastatic carcinoma and angiosarcoma [2]. Several misdiagnosed cases lacked efficient treatments or was only correctly identified after therapy. Kawka et al. [18] reported a case initially diagnosed as bi-lobar intrahepatic cholangiocarcinoma. HEHE definite diagnosis was only established during a follow-up CT scan after the patient received chemotherapy and underwent staged resection (associating liver partition and portal vein ligation for staged hepatectomy (ALPPS)). Another misdiagnosed case was reported by Mucha et al. [19] with an initial diagnosis of carcinoma cholangiogenes desmoplasticum based on needle biopsy. Chemotherapy was first provided for 2 years inefficiently before a further laparoscopic biopsy was carried and HEHE diagnosis was established. Treatment plans were then changed to liver transplant.

The high rate of misdiagnosis created an urgent need to develop accurate diagnostic markers able to identify HEHE patients at early stages. Currently, HEHE diagnosis counts on imaging findings and immunohistochemical staining (IHC) after biopsy. Similar to clinical symptoms, HEHE imaging features are not specific and can rarely lead to definite diagnosis. Nonetheless, several changes have been reported and associated with this type of tumor such as the lollipop sign in magnetic resonance imaging (MRI) and computed tomography (CT) scans. This imaging sign is due to the blocking of branches of portal or hepatic veins by HEHE lesions and is observed as a mass-bar shape resampling a lollipop [9]. The rarity of this sign in other tumors makes it a good representative of HEHE imaging appearance [20]. Other typical alterations such as capsular retraction and target sign have also been associated with HEHE and proposed as specific imaging features [21]. Regardless to the advances in imaging techniques and observations in relevant cases, a definite diagnosis of HEHE is rarely achieved without lesion biopsy and IHC staining. Generally, three different types of cells are observed in HEHE smears: epithelial, dendritic and intermediate cells, with clear expression of biomarkers such as factor VIII antigen (99%), CD34 (94%) and CD31 (86%) [9]. Nonetheless, the specificity of some markers is questionable since expression of markers like CD34 is a shared feature among the majority of vascular tumors [11]. A panel of markers (factor VIII, CD34, CD31, D2-40, Pan-cytokeratin, HepPar-1 and Mucicarmine) can potentially help in differentiating HEHE from other malignancies like HCC, metastatic carcinoma, angiosarcoma and cholangiocarcinoma [2]. Studies also suggested vascular endothelial growth factor (VEGF) as a potential diagnostic marker, considering the vascular nature of HEHE, and proposed anti-VEGF therapy as main or adjuvant treatment for unresectable tumors [22]. As useful puncture biopsy is in HEHE diagnosis, it is important to keep in mind the chances of failure [23] or false negative results (up to 10% [24]) .

Another potential reason for HEHE misdiagnosis is the simultaneous presence of other liver conditions or diseases. HEHE has already been reported in patients with viral hepatitis or liver cirrhosis [25, 26]; however, when HEHE is present with other resembling malignancies like HCC it can be easily misdiagnosed or even overlooked. The typical characteristics and diagnostic biomarkers of tumors like HCC can mask the usually asymptomatic atypical presence of HEHE [27, 28]. 

### The etiology of HEHE

The etiology of HEHE remains unclear since the exact role of potential risk factors is ambiguous. Nonetheless, several risk factors have been associated with HEHE in certain populations. The regular use of oral contraceptives (OC), hepatic diseases (viral hepatitis, alcohol induced damage, primary biliary cholangitis, etc.) and toxic exposure have been reported [16]. Of which, the use of OC attracted a lot of attention. The importance of exploring a link between OC regular intake and HEHE development comes from the high incidence of this tumor in mid-age women (the common population of HEHE patients [1]). Identifying such a link can explain the incidence rates and thus leads to better customized preventive and therapeutic strategies. Suggestions go back to the 1980s when our knowledge regarding EHE in general and HEHE in specific was still limited. In one of the early reports, Dean et al. [29] raised a concern when they identified five female HEHE patients with a mean age of 33 years, all of whom had a long history (4 to 7 years) of OC use. Regardless to sharing such a history, the five patients differed in treatment plans and prognosis. Few years after, Kelleher et al. [30] reported similar findings when four out of six investigated female patients shared a history of OC. Single-case reports also presented such a role of OC as the long-term application (15 years) in one patient led to suspicion of hemangioma when a hepatic lesion was identified and accompanied with unspecific symptoms [31]. 

Another proposed risk factor is vinyl chloride. The ability of this toxic substance to induce HEHE has been reported, especially by older studies, in patients with direct access or at high risk of relevant exposure due to profession [32, 33]. Such findings emphasize the sensitivity of the liver to toxic substances and the extent of damage caused by toxic exposure.

The involvement of other liver conditions in HEHE development has been suspected. Excessive alcohol consumption, hepatic trauma, and viral hepatitis (B and C) have all been mentioned. However, no significant link has been established [2] probably due to the wide presence of such conditions in patients, the general rarity of HEHE, and the complicated involvement of such conditions in the hepatic microenvironment and mechanisms.

### The potential mechanism of HEHE

Similar to its etiology, the mechanism of HEHE is still unclear. Nonetheless, a certain role of genetic alterations has been identified. The acknowledgment of EHE genetic classification by World Health Organization (WHO) further solidified the importance of genetic profiling in this type of tumor. Mainly, two genetic alterations have been described, WWTR1–CAMTA1 and YAP1-TFE3 gene fusions [34]. WWTR1–CAMTA1 fusion refers to the translocation between chromosomes 1 and 3 which activates tumor initiation [35]. This fusion is considered as a genetic marker for EHE since it has been reported in 90% of patients (and no other EHE-mimicking tumors) [36] and translates into nuclear expression of CAMTA1 that has been observed in 85% of HEHE cases [37]. Generally, WWTR1 mutations are oncogenic since they alter certain signaling inhibition (like Hippo pathway) and result in WWTR1 retention in the nucleus rather than the normal translocation of this transcriptional co-activator to the cytoplasm [38]. In addition to its diagnostic value, WWTR1–CAMTA1 fusion has been used to prove the monoclonal nature of HEHE in multifocal cases suggesting a monoclonal metastatic implant rather than multiclonal synchronous oncogenesis [39]. The significance of WWTR1–CAMTA1 fusion in EHE development in general was further validated by Seavey et al. [40] who was the first to successfully create EHE animal model through conditional WWTR1–CAMTA1 knock-in. The genetic intervention resulted specifically in EHE lesions similar to those in human patients in regards to clinical, histological, genetic and immunohistology characteristics. Such results magnify the significance of this genetic fusion in EHE development, address the lack of EHE animal models needed in relevant research and further present WWTR1–CAMTA1 fusion as a genetic marker of EHE.

In addition to WWTR1–CAMTA1 fusion, a less-encountered fusion (YAP1-TFE3) has also been reported. Studies observed features like vaso-formative foci and histiocytoid appearance with voluminous cytoplasm in HEHE with YAP1-TFE3 fusion [41]. TFE3 alterations were also associated with morphologic characteristics such as solid tumor growth, enhanced formation of vascular lumens and lack of stroma [42]. 

Other than genetic profiling, microRNA (miRNA) alterations have also been investigated to further explain HEHE etiology and mechanism. Morishita et al. [43] observed both upregulation (14 miRNAs) and downregulation (93 miRNAs) patterns in HEHE tissues suggesting that certain miRNAs can serve as biomarkers for this tumor.

The established genetic profiling and classification of HEHE patients are extremely helpful when diagnosing this rare tumor. However, the current application of such findings is limited to diagnosis and the relation between such profiling and therapeutic efficacy or prognosis is still unclear. Basing further research (especially basic research and cellular/animal model experiments) on such genetic background might be the missing step to clarify the exact mechanism of HEHE and thus customize treatment strategies.

### The treatment of HEHE

Current HEHE treatments lack a definite guideline due to the rarity of this disease and the inconsistency of clinical course and therapeutic outcome [2]. Nonetheless, several options are provided based on HEHE type, lesions’ number and site, presence of metastasis, patients’ sensitivity and even preferences. Treatment’s options mainly fall into two categories: surgical and non-surgical and vary from traditional resections and transplant to novel immunotherapy and target therapy [44]. 

### Surgical treatments

Surgical approaches (radical resection and liver transplant) are still the first recommended choice when available. Such approaches have been investigated in many studies and associated with enhanced outcome and prognosis compared to non-surgical options. In a retrospective analysis of surveillance, epidemiology, and end results (SEER) database which included 353 HEHE patients [45], Chahrour et al. reported 86.6% one-year overall survival (OS) rate and 75.2% five-year OS rate in patients who underwent surgery compared to 61% and 37.4% in non-surgery group, respectively. Similar results were reported by Grotz et al. [46] who analyzed one, three and five-year OS and disease-free survival (DFS) rates after different treatments (resection group: OS:1-y: 100% / 3-y: 86% / 5-y: 86%, DFS: 1-y: 78% / 3-y: 62% / 5-y: 62%; transplant group: OS:1-y: 91% / 3-y: 73% / 5-y: 73%, DFS: 1-y:64% / 3-y: 46% / 5-y: 46%). The option of liver resection is favorable in HEHE (when possible) considering the general absence of other underlying hepatic conditions (such as liver cirrhosis) which usually complicates surgical approaches in other hepatic malignancies. In addition, resection procedures are usually simpler than transplants and eliminate potential risks of organ failure, prolonged surgeries and hospital-stays [44]. Thus, radical resection is usually the golden option for HEHE. However, liver resections are only recommended when a radical elimination of lesions is possible. Thus, the majority of HEHE patients do not have the chance to benefit from this treatment as they are diagnosed with multi-nodular or diffused HEHE, or even burdened with the presence of extrahepatic metastasis [47]. Furthermore, the risks of post-resection aggressive recurrence, metastasis and unpredictable prognosis still exist [48]. Therefore, relevant factors and patients’ characteristics should be considered thoroughly before surgery. Nonetheless, the enhanced survival and desired outcome associated with liver resections made it a favorable option, with cases of decades-long recurrence-free and metastasis-free being reported [49, 50]. 

Liver transplant is another radical surgery recommended for unresectable HEHE such as those with diffuse manifestations. Cases with extrahepatic metastasis can also benefit from this option as metastasis is not considered a contraindication [51]. However, the lack of clear guidelines in HEHE can sometimes affect the availability and chances of successful transplant [44]. As mentioned, liver transplant has been associated with desired outcome and prolonged survival with short and long-term survival rates as high as 100% and 71.3%, respectively [52]. Nonetheless, the risk of recurrence after the transplant still exists; thus, adjuvant therapies are also recommended after transplant to ensure a better outcome and prevent graft failure or recurrence [53]. In addition to common post-transplant complications (such as graft failure, infection and liver disfunctions), this radical treatment is challenged by the sever insufficiency of donor organs and the long wait-lists. However, HEHE patients are eligible in some countries to receive “exception points” allowing a faster access to available livers and thus shorter pre-operative waiting periods [54]. 

Considering the risks affiliated with surgical treatments and the relatively low malignancy of HEHE, suggestions of observation rather than intervention in some non-aggressive cases have been proposed [11]. Thomas et al. who retrospectively investigated 50 cases of HEHE suggested that an initial observation period may be beneficial and can provide a chance to better understand the behavior of the tumor to determine the right therapeutic approach rather than rushing into surgery or chemotherapy [55]. The encountered cases of stable disease in patients not receiving any treatment or waiting for transplants also supported the notion that surveillance might be sufficient for selective cases of HEHE [56]; however, cases of rapid development leading to liver failure and death have also been reported [57]. Therefore, the decision of surveillance and observation should not be taken lightly as it comes with the potential risks of rapid deterioration and metastasis.

### Non-surgical treatments

A variety of non-surgical options have been explored either as main strategies or adjuvant therapies . Although reported efficacy differs greatly among relevant studies, the majority are faced with the challenges of insufficient numbers of included patients and lack of prospective well-controlled designs.

Similar to other hepatic malignancies, the role of chemotherapy and radiotherapy (RT) in HEHE has been investigated. Mo et al. [58] reported a long-term stability after four combined chemotherapy courses in a HEHE patient with lung metastasis who was followed-up for 13 years, suggesting potential benefits of chemotherapy in cases with extrahepatic lesions. However, the efficacy of chemotherapy is far from guaranteed with studies reporting a lack of response and rapid development of tumor after chemotherapeutic interventions [59]. In addition, chemotherapy is only suggested as a stabilizing treatment rather than radical and usually leads to several adverse events and refractory responses. However, the application of transarterial chemoembolization (TACE) in cases with extrahepatic metastasis led in some studies to better survival and reduced rates of morbidity [60], which is similar to the expected efficacy and application of conventional chemotherapy in other sarcomas such as soft tissue sarcoma (STS) [61] .

As for RT (including Stereotactic body RT, SBRT), it is usually suggested as a complementary therapy to surgical approaches or an alternative when surgery is not a valid option [60]. Similar cases can also benefit from therapeutic options such as ablation (radiofrequency (RFA), microwave (MWA)). The efficacy and safety of such approaches have been supported by relevant reports; however, further prospective investigation is required to draw a certain conclusion [62]. 

The general lack of optimal efficacy created a need to explore novel non-surgical treatments, such as anti-angiogenic therapy. Considering the vascular nature of HEHE development, inhibiting relevant factors like VEGF were suggested as potential treatments for this tumor with many studies investigating the therapeutic effects of different anti-angiogenic agents [9]. Thalidomide is a leading example. Salech et al. [63] reported that thalidomide stopped disease progression in a 40-year-old HEHE patient for 109 months with well tolerance and minimum toxicity. Another long-term efficacy was reported by Raphael et al. [64] in a patient with lung metastasis, the patient had a stable disease with minimal adverse events for seven years. In addition to thalidomide, sorafenib has also been investigated. Kobayashi et al. [65] found sorafenib to be effective when combined with chemotherapy as it led to partial response for 60 months of treatment (first in combination then as a monotherapy). Studies also reported promising results of other agents like lenalidomide [66], pazopanib [67], lenvatinib [68], sunitinib malate [69], paclitaxel and bevacizumab [70]. The combination of bevacizumab and other agents (such as Paclitaxel) showed in several reports promising results including disease control and partial response [70, 71]. Bevacizumab also resulted in a long-term maintenance of efficacy after effective chemotherapy [70]. 

Recently, Interferon alpha-2B (IFN-a 2b) has been getting more attention in research for a promising role in HEHE treatment considering the efficacy of this immunotherapeutic agent in cancer treatment in general. Kayler et al. [72] first reported that IFN-a 2b led to regression in metastatic lesions and symptom relief in a HEHE patient who suffered from recurrence and metastasis after liver transplant. Nonetheless, the patient died due to graft rejection. Better results were reported by Galvão et al. [47] in a HEHE patient with three hepatic lesions. The patient used IFN-a 2b for 9 weeks before qualifying for radical resection then continued IFN-a 2b for an extra week after surgery. The treatment combination was successful as no recurrence or metastasis was observed. Studies with larger patients’ number confirmed the efficacy of IFN-a 2b compared to other treatments. Liu et al. [73] investigated 50 HEHE patients, six of which used IFN-a 2b. Of the six patients, four showed partial response, one complete response and one stable disease. In another study that included 42 HEHE receiving IFN-a 2b treatment [74], rates of partial response, complete response, and stable disease were 47.6%, 4.8% and 28.6%, respectively, with five-year OS and progression-free survival (PFS) as high as 97.2% and 62.3%. Additionally, no grade 3 adverse events were observed. Such results highlight the potential of IFN-a 2b in HEHE treatment due to its convenience , limited side effects and efficacy in treatment combination. However, the exact mechanism in which IFN-a 2b acts is still unclear. Only limited number of studies investigated IFN-a 2b in the hepatic microenvironment and none were in HEHE models. Suggested mechanisms included activation of intrinsic hepatic NK cells [75], inhibition of cyclooxygenase-2 (COX-2) and VEGF expression [76] and decreasing cholesterol levels to induce cellular apoptosis [77]. 

Sirolimus is another immunotherapeutic agent attracting attention with its promising potential. This mammalian target of rapamycin (mTOR) inhibitor has shown promising results in EHE patients in general. In a study by Engel et al. [78], six pediatric EHE patients were treated by sirolimus either as first treatment or later during their therapy course. Partial response and stable disease were observed in four patients with well-tolerance and side effects limited to grade 1. Cournoyer et al. [79] who investigated 24 EHE patients reported a mean time to progression of 18.4 months; however, the majority of patients treated with sirolimus (6/8) partial response or stable disease with three cases being responsive for more than 2.5 years. In a case-series analysis with a larger number of patients treated with sirolimus (37 cases) [80], Stacchiotti et al. reported a 10.8% partial response rate and 75.7% stable disease rate. Median OS and PFS were 18.8 months and 13 months, respectively. As promising such results are, a severe lack of investigations focusing on sirolimus efficacy specifically in HEHE patients is still present. 

A variety of novel agents are also being investigated for HEHE treatment. However, more efforts are required to understand the exact mechanisms and appropriate application before clinical application, considering that current studies are mainly single-case reports or with insufficient number of patients.

## Discussion and conclusion

The rarity of HEHE and atypical manifestation led to clear limitations in our understanding of its mechanism. Furthermore, the low incidence of HEHE created a lack of relevant researches and a trend of single dimensional investigating strategies. To evaluate the current research efforts investigating HEHE, we carried a simple review of available articles on PubMed platform (https://pubmed.ncbi.nlm.nih.gov). Using the key words “Epithelioid Hemangioendothelioma and Liver”, we checked for articles published before November, 2022. Initially, a total of 693 articles were available. However, after reviewing articles’ titles and abstracts and excluding irrelevant articles, review articles and non-English articles, only 241 articles were relevant and further reviewed. The publication of relevant articles started in the early 1980s, however, an obvious increase in publication was only observed between 2020 and 2022. Compared to researches of other hepatic malignancies, a total of 241 articles over a period of 40 years is considered as a severe lack of research. Furthermore, a total of 131 articles (54%) were single-case reports with only 10 articles (4.15%) including more than 100 cases. The vast majority of articles focused on describing the manifestation and clinical features in included patients, or evaluating the efficacy of diagnostic tools and therapeutic options. The retrospective nature of relevant research was also apparent. Although such studies helped in developing our understanding of HEHE; however, the inconsistency in reported results, the limited number of included cases and the lack of prospective designs prevented the ability to develop clear guidelines for the treatment of HEHE or accurate classification method for patients.

In addition, the rarity of EHE heavily limited our knowledge of this disease in specific groups of patients such as pediatric population. With a very limited number of relevant studies, pediatric EHE has been characterized by a female predominance, asymptomatic multi-organ manifestation, and specific genetic (WWTR1-CAMTA1 fusion) and IHC (CD31+, CD34+, factor VIII+) profiles, which are similar to what is known about this disease in adults [81]. As for treatment, surgery is the favorable option in resectable cases. However, strategies in unresectable cases are under-investigated and lack supporting evidence [82].

Therefore, HEHE still creates a challenge in clinical practices. Many obstacles are faced when providing medical care due to the unique challenges brought by this tumor. The lack of clear etiology and mechanism of HEHE creates a challenge in identifying high-risk patients or establishing an early-stage diagnosis. In addition, HEHE rarity, heterogenicity, atypical and asymptomatic manifestation all increase the risks of misdiagnosis. Such challenging profile translated into a sever lack of relevant research both in number and quality. That created an increased difficulty to establish a clear guideline for treatment plans. Currently, radical surgery is the golden option; however, only a small number of HEHE patients are eligible for surgery due to HEHE manifestation, extrahepatic metastasis and lack of organ donors. Non-surgical approaches are being investigated with some treatments, like IFN-a 2b, showing a great potential. However, the rarity of HEHE and lack of prospective research limit the clinical application and value of potential therapies.

Based on the discussed literature and reported outcome of different treatments, surgical resection can be the first choice for early-stage resectable cases. A liver transplant can be considered for multiple-lesion manifestation when no metastasis is present. However, therapeutic combinations of locoregional approaches (such as TACE and RFA) and systemic therapies (such as interferon and bevacizumab) may be beneficial for late-stage or post-surgical recurrent tumors. Regardless to the treatment of choice, a close timely monitoring should be carried to evaluate the efficacy and allow adjustments at early stage of progression. Thus, observation is mainly suggested for long-term stable cases or when the behaviour of tumor is yet to be defined.

Further research is needed to improve our understanding of HEHE especially in specific populations like childhood patients. Enhancing our knowledge of this rare tumor may improve the awareness in clinical practices and help doctors to suspect and identify cases as early as possible. Developing stronger research strategies, larger funding and cooperation between centers can all accelerate the process to establish diagnosis and treatment guidelines able to regulate and enhance the medical care provided to HEHE patients.

## Electronic supplementary material

Below is the link to the electronic supplementary material.

Supplementary Material 1

Supplementary Material 2

## Data Availability

No datasets were generated or analyzed during the current study as it is a review article. Further inquires, if needed, are available from the authors upon request.

## Abbreviations

ALPPS: Associating liver partition and portal vein ligation for staged hepatectomy
COX-2: Cyclooxygenase-2
CT: Computed tomography
DFS: Disease-free survival
EHE: Epithelioid hemangioendothelioma
HCC: Hepatocellular carcinoma
HEHE: Hepatic epithelioid hemangioendothelioma
IFN-a 2b: Interferon alpha-2B
IHC: Immunohistochemical staining
miRNA: Micro ribonucleic acid
MRI: Magnetic resonance imaging
mTOR: Mammalian target of rapamycin
OC: Oral contraceptives
OS: Overall survival
PFS: Progression-free survival
RT: Radiotherapy
SBRT: Stereotactic body radiotherapy
SEER: Surveillance, epidemiology, and end results
VEGF: Vascular endothelial growth factor
WHO: World health organization
