# Supplementary material for: The challenges of hepatic epithelioid hemangioendothelioma: the diagnosis and current treatments of a problematic tumor
Source: Orphanet J Rare Dis. 2024 Nov 30;19:449. doi: 10.1186/s13023-024-03354-z (PMC11608485; doi:10.1186/s13023-024-03354-z)
Supplement: Supplementary file 1 — Supplementary Material 1 [file 13023_2024_3354_MOESM1_ESM.docx]

**Cover letter:**

Dear editors,

We present to you herewith a manuscript of a review article titled “the challenges of hepatic epithelioid hemangioendothelioma: the diagnosis and current treatments of a problematic tumor.” to be considered for publication in your respected journal.

As indicated in the title, the article focuses on hepatic epithelioid hemangioendothelioma (HEHE), a malignant vascular tumor of the liver known for its rarity with an incidence of only 1/1000,000. Regardless to decades passing since its discovery, HEHE still creates a clinical dilemma due to its challenging clinical manifestation, lack of relevant research and treatment guidelines. In this review, we address the unique profile and current clinical challenges that complicate the diagnosis and treatment strategies of HEHE and thus limit the quality and efficacy of clinical care provided to patients. We also focus on current therapeutic strategies and the limitations influencing the choice of treatment plans.

Key challenges have been included in this review such as the unclear etiology and mechanism, the nonspecific clinical manifestation and misdiagnosis, the insufficiency of relevant research and more importantly the lack of treatment guidelines and limitations of current treatment options.

Considering the rarity of HEHE and the lack of awareness of this disease in the majority of clinical practices, we believe that this review article falls in line with the aim of your respected journal and will be of great interest and use to your reader.

We would also like to emphasize that the content of this work has not been published or submitted elsewhere and that all authors have read and approved the submission and declare no competing interests.

Finally, we deeply appreciate your consideration and we hope that this manuscript will meet your requirements for publication.

Corresponding authors,

Zhiying Yang,

Department of Hepatobiliary Surgery, China-Japan Friendship Hospital, Beijing, China.

Tel: +86-10-84205054; E-mail: [yangzhy@aliyun.com](mailto:yangzhy@aliyun.com)

Yongliang Sun,

Department of Hepatobiliary Surgery, China-Japan Friendship Hospital, Beijing, China.

<Tel:+86-13581884881>; E-mail: sunyunliang1982@homail.com
