# Supplementary material for: The challenges of hepatic epithelioid hemangioendothelioma: the diagnosis and current treatments of a problematic tumor
Source: Orphanet J Rare Dis. 2024 Nov 30;19:449. doi: 10.1186/s13023-024-03354-z (PMC11608485; doi:10.1186/s13023-024-03354-z)
Supplement: Supplementary file 2 — Supplementary Material 2 [file 13023_2024_3354_MOESM2_ESM.doc]

Dear editors,

We first want to thank you for completing the review process of our manuscript. As requested, we revised the manuscript to meet the requirements of both reviewers. We tried to the best of our ability to address all points raised and enhance the quality of the manuscript while keeping the integrity of the topic discussed.

Here, we include a point-to-point response to answer reviewers’ concerns and explain the enchantments applied to the manuscript.

**Response to Reviewer #1:**

Thank you for the comments and constructive suggestions. Your comments helped us realize few limitations of the manuscript that we addressed in the revised version.

**Comment 1:**

“While the summaries of molecular biology of diagnosis and non-surgical and surgical treatments are helpful, the structure of the piece, based the three challenges, all of which are generalisable to most very rare diseases lead to discussions which are too general, overlap and are difficult to follow. The conclusions as summarised above are also general and negative.”

Response:

While the original structure (basing the narrative on three challenges) was meant to avoid the typical narrative of similar reviews, it might have created an unnecessary confusion and a tendency to generalize content. Your comment helped us to realize the need to adopt more conventional narrative. Thus, we completely changed the structure of manuscript, especially the discussion section to make it easier to follow while adding depth to the discussion of the topic.

**Comment 2:**

”As a paediatric hepatologist I have only encountered this tumour twice in children where it is even rarer than in adults. Nevertheless to include a brief paragraph to acknowledge the analogous paediatric tumours and state that they are similar or dismissing them as completely different would be helpful.”

Response:

Based on the request, a brief discussion of pediatric HEHE has been added, acknowledging this specific population of patients and drawing a simple comparison with HEHE in adults.

**Comment 3:**

If they wish to re-submit the article, the authors should re-write it in a more conventional format defining the disease entity, examining its associations, presentations, diagnosis and sub-diagnoses if relevant, its treatments; effective and otherwise, and outcomes, acknowledging the lack of consistency in the literature and including in their conclusion a proposed evidence based algorithm for treating patients from their presentation. The evidence would naturally have weaknesses but would be the best that could be constructed in the circumstances. Paediatric patients would probably be excluded.

Response:

As mentioned, the structure of the manuscript has been revised, following the suggested format. An algorithm of treatment has also been added to the discussion section which we believe has enriched the discussion of this point.

**Comment 4:**

In addition the use of English is non-Native and sometimes colloquial rather than scientific. A review of the text to consider the use of English and spelling would help in understanding the complexity of the evidence.

Response:

A second round of revision and editing has been carried to enhance the quality of the manuscript and address the limitations mentioned in the comment.

**Response to Reviewer #2:**

Thank you for the comments and suggested references.

**Comment 1:**

Radiofrequency ablation (RF), transarterial chemoembolization (TACE), SBRT are also possible treatment options for this tumor. These should be mentioned in the nonsurgical treatments section.

Response:

Relevant section has been added to the manuscript to address the suggested treatments.

**Comment 2:**

Chemotherapy regimens mentioned in the nonsurgical treatments section are outdated. References to the use of IFN are 20-year-old articles. Anti VEGF tyrosine kinase inhibitors, bevacizumab and chemotherapy protocols for sarcoma are current treatment options. This section of the article should be majorly changed.

Response:

The original manuscript already had a section addressing anti-VEGF approaches, and references to the use of IFN also included recent studies and not only 20-year-old articles. Nonetheless, the rarity of HEHE articles indeed influenced this issue. Therefore, further content has been added, and major changes of the treatment section have been carried to provide the needed clarity. Further discussion of chemotherapy and bevacizumab has also been added to enrich the content .

Corresponding authors,

Zhiying Yang,

Department of Hepatobiliary Surgery, China-Japan Friendship Hospital, Beijing, China.

Tel: +86-10-84205054; E-mail: [yangzhy@aliyun.com](mailto:yangzhy@aliyun.com)

Yongliang Sun,

Department of Hepatobiliary Surgery, China-Japan Friendship Hospital, Beijing, China.

[Tel:+86-13581884881](tel:+86-13581884881); E-mail: sunyunliang1982@hotmail.com
